# Anti-pulmonary fibrosis activity analysis of methyl rosmarinate obtained from *Salvia castan*ea Diels *f. tomentosa* Stib. using a scalable process

**DOI:** 10.3389/fphar.2024.1374669

**Published:** 2024-06-04

**Authors:** Li Ma, Chuntong Liu, Yuxiang Zhao, Mengke Liu, Yunyi Liu, Huachang Zhang, Shude Yang, Jing An, Yuheng Tian, Yinchuan Cao, Guiwu Qu, Shuling Song, Qizhi Cao

**Affiliations:** ^1^ Binzhou Medical University, Shandong, China; ^2^ Department of Edible Mushrooms, School of Agriculture, Ludong University, Shandong, China; ^3^ Division of Infectious Diseases and Global Health, School of Medicine, University of California San Diego (UCSD), La Jolla, CA, United States; ^4^ Shandong Engineering Research Center for Functional Crop Germplasm Innovation and Cultivation Utilization, Shandong, China

**Keywords:** pulmonary fibrosis, MR, TGF-β/Smad, MAPK, RA

## Abstract

Pulmonary fibrosis is a progressive, irreversible, chronic interstitial lung disease associated with high morbidity and mortality rates. Current clinical drugs, while effective, do not reverse or cure pulmonary fibrosis and have major side effects, there are urgent needs to develop new anti-pulmonary fibrosis medicine, and corresponding industrially scalable process as well. *Salvia castanea* Diels f. *tomentosa* Stib., a unique herb in Nyingchi, Xizang, China, is a variant of *S. castanea*. and its main active ingredient is rosmarinic acid (RA), which can be used to prepare methyl rosmarinate (MR) with greater drug potential. This study presented an industrially scalable process for the preparation of MR, which includes steps such as polyamide resin chromatography, crystallization and esterification, using *S. castanea* Diels f. *tomentosa* Stib. as the starting material and the structure of the product was verified by NMR technology. The anti-pulmonary fibrosis effects of MR were further investigated *in vivo* and *in vitro*. Results showed that this process can easily obtain high-purity RA and MR, and MR attenuated bleomycin-induced pulmonary fibrosis in mice. *In vitro*, MR could effectively inhibit TGF-β1-induced proliferation and migration of mouse fibroblasts L929 cells, promote cell apoptosis, and decrease extracellular matrix accumulation thereby suppressing progressive pulmonary fibrosis. The anti-fibrosis effect of MR was stronger than that of the prodrug RA. Further study confirmed that MR could retard pulmonary fibrosis by down-regulating the phosphorylation of the TGF-β1/Smad and MAPK signaling pathways. These results suggest that MR has potential therapeutic implications for pulmonary fibrosis, and the establishment of this scalable preparation technology ensures the development of MR as a new anti-pulmonary fibrosis medicine.

## 1 Introduction

Pulmonary fibrosis is an interstitial lung disease that often presents as a heterogeneous, chronic, progressive end-stage development of various acute and chronic lung diseases (Dempsey, 2006; Raghu et al., 2011; Koudstaal et al., 2023). Idiopathic pulmonary fibrosis (IPF) is a severe form of pulmonary fibrosis with unknown etiology. The pathogenesis of pulmonary fibrosis is currently accepted to follow the epithelial cell/extracellular matrix hypothesis, which suggests that repeated and unexplained endogenous or exogenous stimuli lead to lung epithelial cell damage that cannot be repaired by normal processes, and fibroblasts in the ECM are activated to assume a myofibroblast morphology and form fibroblast foci with overproduction of the extracellular matrix (ECM) components, foveal lesions, and fibrosis (Ley and Collard, 2013; Camelo et al., 2014; Mei et al., 2021; Zhang et al., 2022). Pirfenidone and nintedanib have been approved by the FDA, the European Medicine agency and more than 75 countries, as both drugs can slow the progression of the disease to some extent (King et al., 2014; Richeldi et al., 2014; Liu et al., 2017; Sgalla et al., 2018; Huh et al., 2023). However, neither of these two drugs can stop or reverse fibrosis, nor do they significantly decrease mortality rates. Lung transplantation (George et al., 2019) remains the mainstay of treatment for pulmonary fibrosis; however, its high surgical risk, high cost and frequency of subsequent transplant rejection are not acceptable to the general public. Therefore, an urgent need exists to develop new medicines for the treatment of pulmonary fibrosis.

Rosmarinic acid (RA), which can be used for preparation of methyl rosmarinate (MR), is a water-soluble natural phenolic compound mainly found in various plants such as the Lamiaceae, Lithospermum, Cucurbitaceae, Tiliaceae, and Umbelliferae families. *Salvia castanea* Diels f. *tomentosa* Stib. is a variant of *S. castanea.*, a unique herb in Nyingchi, Xizang, China, used as a substitute for *Salvia miltiorrhiza* Bge. in Tibet. Its main active ingredient is RA. RA has been reported to possess potent and wide-ranging medicinal properties, including anti-inflammatory (Chu et al., 2012; Liang et al., 2016), antibacterial (Slobodníková et al., 2013), antiviral (Dubois et al., 2008), antitumor (Saiko et al., 2015), antioxidant activities (Osakabe et al., 2002; Kim et al., 2005; Cai et al., 2022), and anti-pulmonary fibrosis activities (Bahri et al., 2017; Zhang J. et al., 2020; Bahri et al., 2021). A related RA derivative, MR, can be obtained by esterification of RA with methanol under the catalysis of hydrochloric acid, is structurally similar to RA, but differs in the esterification of the side chain carboxyl groups, imparting a greater lipid solubility. Current activity studies on MR have mainly demonstrated its antitumor, antibacterial, antioxidant, anti-glycosylation, and anti-inflammatory effects (Lin et al., 2013; So et al., 2016; Nam et al., 2019; Gul et al., 2023). However, its anti-pulmonary fibrosis effects and mechanisms have not been described. In this study, we investigated the anti-pulmonary fibrosis effects of MR in terms of potential modulation of pulmonary TGF-β1 and MAPK signaling.

TGF-β1 is known to play an important regulatory role in cell proliferation, differentiation, phenotypic regulation, organ fibrosis, wound healing, tumorigenesis, and metastasis. It is considered to serve as the linchpin in the initiation and development of pulmonary fibrosis (Djudjaj and Boor, 2019; Li et al., 2023). TGF-β1 binds to cell membrane receptors to trigger a cascade response by activating downstream Smad signaling and non-Smad signaling, which mediate the development of pulmonary fibrosis (Andugulapati et al., 2020). Therefore, targeting TGF-β-induced fibroblast activation may be a potential approach for the treatment of IPF.

In this study, we established a scalable process for the preparation of MR using *S. castanea* Diels f. *tomentosa* Stib. as the starting material. Bleomycin (BLM)-treated mice and TGF-β1-stimulated mouse fibroblast L929 cells were used to explore the effects of MR on lung fibrosis. The experimental data showed that MR significantly ameliorated BLM-induced mouse lung fibrosis, and it inhibited TGF-β1-induced L929 cells activation and collagen I and III accumulation, MR demonstrates a stronger effect than RA in two aspects. MR treatment also effectively inhibited the proliferation and migration of TGF-β1-induced L929 cells and promoted their apoptosis via TGF-β/Smad and MAPK cell pathway. Our findings indicate that MR has the potential for development as an anti-pulmonary fibrosis medication.

## 2 Materials and methods

### 2.1 Cell culture and treatment

The mouse fibroblast L929 cell line was obtained from the Stem Cell Bank of the Chinese Academy of Sciences (Shanghai, China) and cultured in RPMI-1640 medium supplemented with 10% fetal bovine serum (FBS, Gibco, United States) and antibiotics (100 U/mL penicillin and 100 μg/mL streptomycin) in a humidified incubator at 37°C in the present of 5% CO_2_. When the cells reached to approximately 50%–70% confluence, they were treated with different concentrations of drugs. Dimethyl sulfoxide (DMSO) was used as a vehicle control. All cell lines were mycoplasma-free and characterized by the Cell Bank of the Chinese Academy of Sciences.

### 2.2 Reagents and antibodies

Horseradish peroxidase (HRP)-coupled goat anti-rabbit and HRP-coupled goat anti-mouse antibodies were purchased from Zhongshan Jinqiao Biotechnology Co. Ltd (Beijing, China); TGF-β1 was purchased from Biolegend (United States). CCK-8 assay kit was purchased from Nippon Tongren Institute of Chemical Research (Japan); Annexin-V (FITC) apoptosis detection kit was purchased from Immuno Way Biotechnology (United States); BLM was purchased from BioChemPartner (Shanghai, China); H&E staining kit was purchased from Koolabo Technology (Beijing, China); Sirius red staining kit was purchased from Solarbio (Beijing, China). The following primary antibodies were purchased from Cell Signal Technology (United States): anti-vimentin (#5741T), anti-snail (#3978S), anti-Smad2 (#5229T), anti-p-Smad2 (#18338T), anti-Smad3 (#9523T), anti-p-Smad3 (#9520T), anti-ERK (#4695S), anti-JNK (#9252T), anti-p-JNK (#4668S), anti-p38 (#9212S), and anti-p-p38 (#4511T); anti-p- ERK (ab201015) was purchased from Abcam (United Kingdom); anti-GAPDH (10494-1-AP) was purchased from Proteintech (Wuhan, China).

### 2.3 Preparation of methyl rosmarinate

*Salvia castanea* Diels f. *tomentosa* Stib. is rich in RA, which can react with methanol under the catalysis of hydrochloric acid to produce MR. A process for preparing MR using *castanea* Diels f. *tomentosa* Stib. as the starting material is developed, which includes steps such as polyamide resin chromatography, crystallization and esterification. The process is as follows:

Dry roots of *S. castanea* Diels f. *tomentosa* Stib. was crushed to the coarsest powder, then soak and extract twice at room temperature with 50% ethanol (adjusted to pH 3 with hydrochloric acid). The first extraction takes 4 h, with a solvent dosage of 20 times the mass of the coarse powder, and the second extraction takes 2 h, with a solvent dosage of 10 times. Filter and merge the extraction solution, remove ethanol under reduced pressure at 65°C, and then perform chromatographic separation on it using polyamide resin as the medium. The operating load is 25 mg RA/mL medium. The elution process is as follows: first, wash 8 column volumes with 25% ethanol (adjusted to pH 3 with hydrochloric acid), discard the effluent, then wash 6 column volumes with 60% ethanol and collect this fraction. Concentrate at 65°C under reduced pressure to about 50 mg RA/mL, filter while hot, let the filtrate stand overnight at room temperature to obtain granular crystals of RA.

Filter and collect RA crystals, and dry them under reduced pressure at 45°C. Take 10 g and dissolve it in 250 mL absolute methanol. Add 2.5 mL hydrochloric acid as a catalyst and react at room temperature for 24 h. After the reaction is completed, the reaction solution is depressurized at 45°C to remove methanol and then dissolved in an appropriate amount of ethyl acetate and washed three times with water. Collect ethyl acetate phase and depressurized at 45°C to remove ethyl acetate, the final product, namely, MR, has a purity of over 98%. During this process, HPLC is used to track and detect RA and MR. The HPLC method is: C_18_ reverse phase chromatography column, detection wavelength of 286nm, mobile phase of 2% formic acid (A)—acetonitrile (B), gradient elution program: 0–15 min, 10% B → 100% B; 15–20 min, 100% B; 20–25 min, 100% B → 10% B; 25–30 min, 10% B.

### 2.4 Structural verification of methyl rosmarinate

The samples obtained above was dissolved in DMSO-*d*_6_ for NMR analysis. Data obtained by Bruker Advance Nero 600.

### 2.5 Cell counting kit-8 (CCK-8) assay

The cytotoxicity of MR and RA on L929 cells was evaluated using the CCK-8 assay. 100 μLL929 cells at a cell density of 1×10^5^ cells per ml were seeded in 96-well plates and incubated overnight. Fresh culture medium containing 0, 10, 20, 40, 80, and 160 μM MR and RA were added to each well and cultured for an additional 48 h. Subsequently, 10 µL CCK-8 was added to each well and incubated for 3 h. Absorbance at 450 nm was detected using a Tecan Infinite M200 Pro multimode microplate reader (Shanghai, China). The IC50 (half inhibitory concentration) values were expressed as the drug concentration that induced 50% cytotoxicity. In addition, the effects of MR on the proliferation of TGF-β1-induced L929 cells were detected using the CCK-8 method described above. Cells were divided into the following groups: a negative blank control, TGF-β1 (5 ng/mL), MR (20 μM) +TGF-β1 (5 ng/mL), MR (40 μM) +TGF-β1 (5 ng/mL).

### 2.6 Animal model and ethics statement

Male C57BL/6 mice (6–8 weeks old) with an average weight of 20 ± 5 g were purchased from Jinan Peng Yue Company. The *in vivo* experiments were approved by the Animal Experimentation Ethics Committee of Binzhou Medical College (2023-371) and conducted in strict accordance with the National Institutes of Health Guidelines for the Care and Use of Laboratory Animals. All mice were maintained on a 12-h light/dark cycle and given *ad libitum* access to food and water. One week after acclimatization, mice were randomly divided into 5 treatment groups (n = 8): saline, BLM, BLM + MR (20 mg/kg), BLM + RA (20 mg/kg), and BLM + PFD (300 mg/kg). Mice were fasted for 3 h, and 5 mg/kg BLM dissolved in saline was sprayed into the lung tissue of mice using a Penn-Century MicroSprayer (Penn-Century, Inc., Pennsylvania, PA) to induce pulmonary fibrosis. The saline group received an equal amount of saline in the same manner. On day 3 after BLM-induced injury, MR or RA dissolved in 1% CMC-Na solution was administered daily by gavage, as close to 1 h as possible, and continued until 28 days later. Pirfenidone was used as a positive control. Both control and model groups received the same dose of 1% CMC-Na solution with the same dosing schedule and route of administration. Body weights of mice were recorded until day 28 when they sacrificed for subsequent experiments.

### 2.7 Hematoxylin-eosin (HE) and sirius red staining

The left lung was ventilated and placed in fresh 4% neutral paraformaldehyde buffer for 24 h at room temperature. After paraffin embedding, 4-μm sections were prepared and stained with H&E to observe the tissue structure. Collagen deposition was detected by Sirius red staining. The severity of interstitial fibrosis in each consecutive region was scored individually and blindly according to the Ashcroft scoring system described by Janine Schnicring et al. (Schniering et al., 2018).

### 2.8 Wound scratch assay

1.5 × 10^4^ cells per well were seeded in 6-well plates and induced with TGF-β1 (5 ng/mL). When cells grew to approximately 80% confluence, scratches were made with a sterile 1 mL pipette tip. After two washes with PBS to remove cell debris, cells were cultured with fresh medium containing MR (0, 20, or 40 μM) in each well. Photographs were taken at 0, 24 and 48 h using an inverted microscope. Scratch area analysis was performed using ImageJ software (National Institutes of Health, Bethesda, MA).

### 2.9 Apoptosis analysis

2 × 10^5^ L929 cells per well were seeded in 6-well plates at. After the cells entered the logarithmic growth phase and were induced by TGF-β1 (5 ng/mL), MR (0, 20, 40 μM) was added for 48 h. After harvesting and washing twice with pre-cooled PBS, the cells were stained with Annexin V-FITC/PI and protected from light for 15–20 min. Cell samples were then immediately analyzed using a flow cytometer (BD Biosciences, Franklin Lakes, NJ, United States).

### 2.10 Western blotting analysis

L929 cells with good growth status were collected, induced by TGF-β1, and then added with (20, 40 μM) MR for 48 h. Target cells were harvested and lysed in RIPA buffer supplemented with phosphatase inhibitors and protease inhibitors for 30 min on ice. The supernatant was collected after centrifugation at 4°C for 15 min at 12,000 rpm, and the protein concentration was determined using a BCA protein analysis kit (Solarbio, Beijing, China). For the extraction of lung tissue proteins, forceps and scissors were cleaned with an alcohol cotton ball, approximately 25 mg of lung tissue was cut and weighed, the tissue was ground by adding lysis solution, and the supernatant was centrifuged to extract the proteins; the lysis and extraction processes were carried out on ice, and the concentration of the proteins was determined by BCA. The cell and tissue protein extracts were subsequently separated by 10%–15% SDS-PAGE, transferred to nitrocellulose membranes (Millipore, Billerica, United States), blocked with 5% skim milk for 2 h, and incubated with the appropriate primary antibodies at 4°C overnight, followed by the incubation with the corresponding secondary antibody at room temperature for 90 min. After adding the electrochemiluminescence (ECL) chromogenic solution for color development (NOVLAND, no.PWB-001S), the target genes were detected using a Tanon 5,200 chemiluminescence imaging analysis system (Shanghai, China). The exposure time was set to automatic exposure. Quantitative analysis was performed using ImageJ software.

### 2.11 Statistical analysis

Each experiment was performed independently and was repeated at least three times. GraphPad Prism 5.0 software package (Graph Pad Software, Inc., La Jolla, CA) was used for all statistical analyses. The t-test was used for comparison between the two groups, and the ANOVA analysis was used for the comparison between multiple groups. Data are expressed as mean ± standard deviation (SD), and *p* < 0.05 is considered statistically significant.

## 3 Results

### 3.1 Preparation and structural validation of MR

**FIGURE 1:**
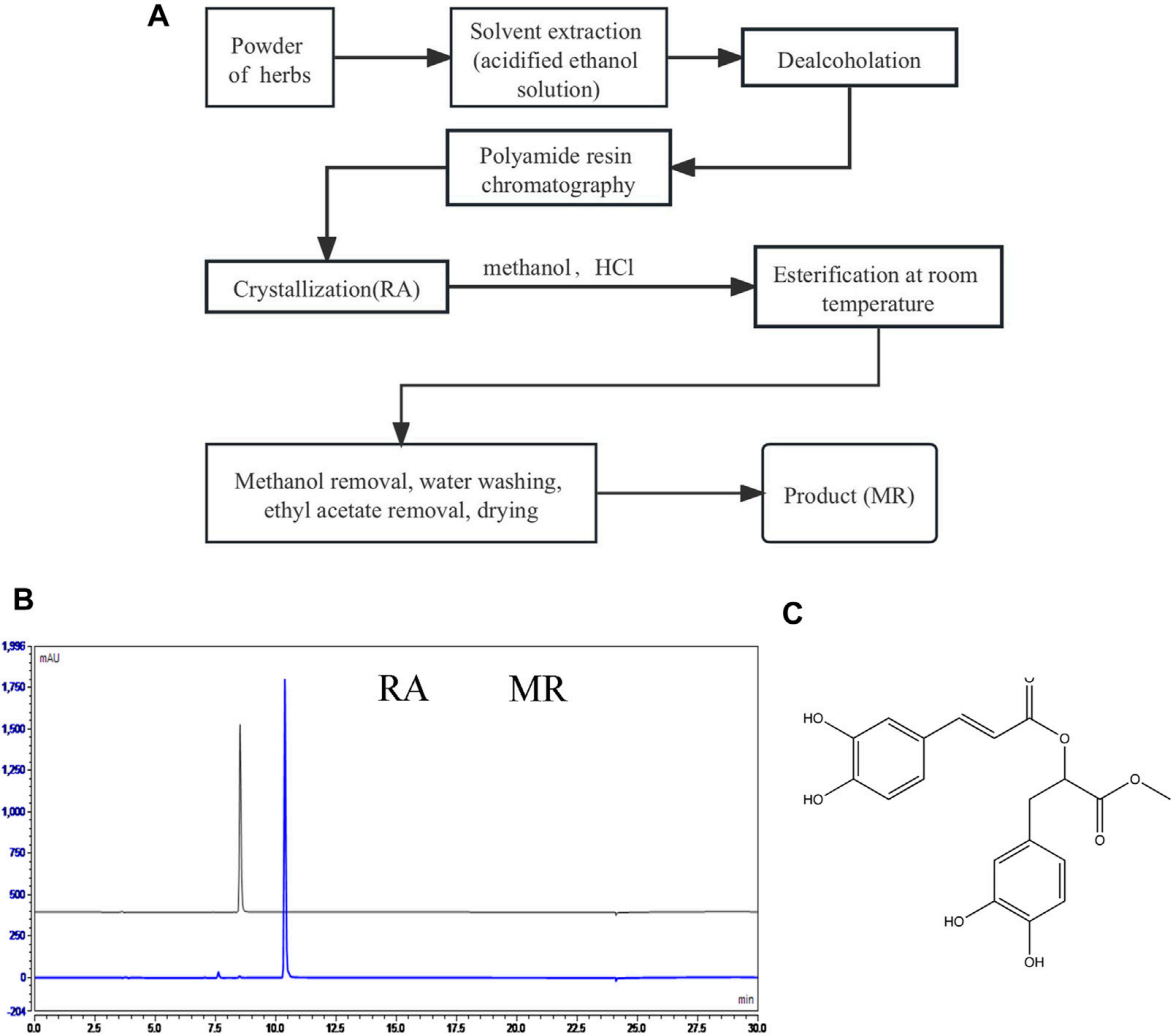
Preparing and identification of MR. **(A)** The process of preparing MR using *castanea* Diels f. *tomentosa* Stib. as the starting material. **(B)** HPLC diagrams of RA and MR obtained by the above process. **(C)** The molecular structure of MR.

The process of preparing MR using *Salvia castanea* Diels f. *tomentosa* Stib. as the starting material including polyamide resin chromatography, crystallization and esterification is shown in Figure 1A, high purity RA and MR obtained through this process and the consumables, equipment, technology and operating procedures used are common in industrial processes. A laboratory scale preparation was carried out according to this process, and 9.1 g of light-yellow oily liquid was obtained with a purity of 98.12% (Figure 1B). The NMR data are as follows:

^1^H NMR (600 MHz, DMSO) δ 9.59 (s, 1H), 9.19 (s, 1H), 8.79 (s, 2H), 7.49 (d, *J* = 15.9 Hz, 1H), 7.07 (d, *J* = 2.2 Hz, 1H), 7.03 (dd, *J* = 8.2, 2.1 Hz, 1H), 6.78 (d, *J* = 8.1 Hz, 1H), 6.67–6.63 (m, 2H), 6.51 (dd, *J* = 8.0, 2.1 Hz, 1H), 6.27 (d, *J* = 15.9 Hz, 1H), 5.13 (dd, *J* = 7.7, 5.1 Hz, 1H), 3.64 (s, 3H), 3.02–2.91 (m, 2H).^13^C NMR (151 MHz, DMSO) δ 170.40, 166.35, 149.21, 146.82, 146.08, 145.46, 144.60, 127.10, 125.75, 122.16, 120.53, 117.14, 116.23, 115.90, 115.44, 113.31, 73.27, 52.45, 36.66.

Based on the above NMR data, it is confirmed that the compound is MR (C_19_H_18_O_8_), and the structure is shown in Figure 1C.

### 3.2 Anti-pulmonary fibrotic effects of MR on TGF-β1- treated L929 cells

**FIGURE 2:**
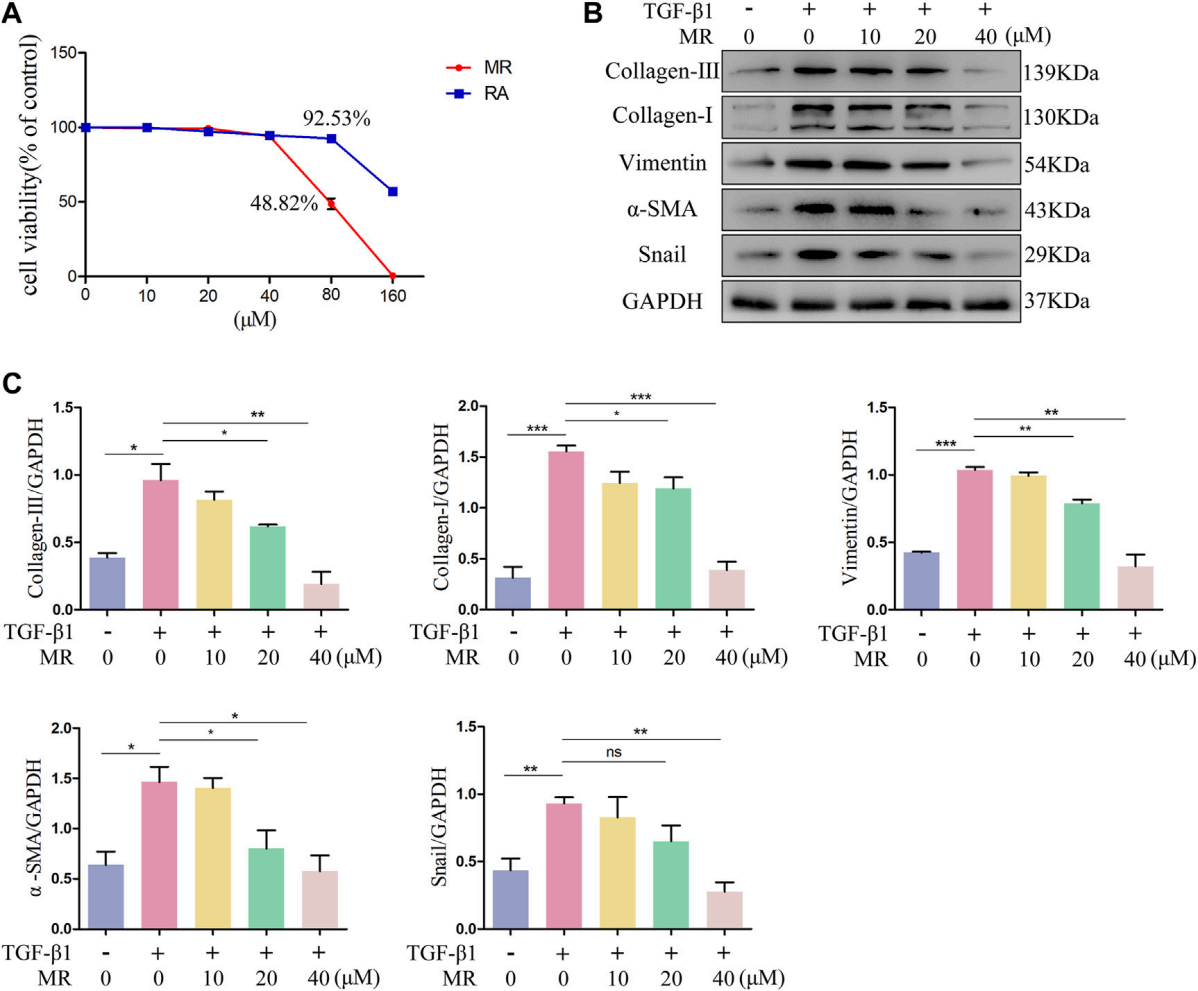
Anti-pulmonary fibrosis effect of MR on TGF-β1-induced L929 cells. **(A)** The cytotoxicity of MR and RA in normal L929 cells was determined using the CCK-8 assay and IC50 values were calculated. **(B)** Analysis of the anti-pulmonary fibrotic effect of MR by Western blots. The expression of collagen I and III, vimentin, α-SMA, and snail in L929 cells were examined after stimulation with TGF-β1. **(C)** Quantitative analysis based on the gray value of each band. Data are expressed as mean ± SD (*p < 0.05, **p < 0.01, and ***p < 0.001).

Mouse fibroblast L929 cell line, possesses characteristics similar to lung fibroblasts in terms of hyperproliferation and activation, and can be induced into myofibroblasts with TGF-β1. TGF-β1 induced L929 cells are commonly used as the cell models of lung fibrosis (Zhang T. et al., 2020; Li T. et al., 2022; Wang et al., 2024). The cytotoxic effects of MR and RA were evaluated by conducting CCK-8 assays on L929 cells exposed to different concentrations (0, 10, 20, 40, 80 and 160 μM) of MR and RA (Figure 2A). The IC50 values for MR and RA in L929 cells were 76.27 μM and 170.7 μM. Respectively. The anti-fibrotic effects of MR were examined by performing the following experiments: after inducing fibrosis in L929 cells with TGF-β1 (5 ng/mL) for 72 h, different concentrations of (0, 10, 20, or 40 μM) MR were added, and the cells were incubated for 48 h. The expression levels of key proteins associated with lung fibrosis in treated cells were then examined by western blotting. The expression of collagen-Ⅰ, collagen-Ⅲ, vimentin, α-SMA, snail was significantly increased after 72 h of TGF-β1 stimulation, and this elevated expression was dose-dependently reversed by exposure to MR (Figures 2B,C), indicating that MR could reduce the deposition of ECM, thereby attenuating the development of pulmonary fibrosis. For convenience, we chose to use 0, 20, and 40 μM as the drug concentrations for subsequent experiments.

### 3.3 MR attenuated the BLM induced pulmonary fibrosis

**FIGURE 3:**
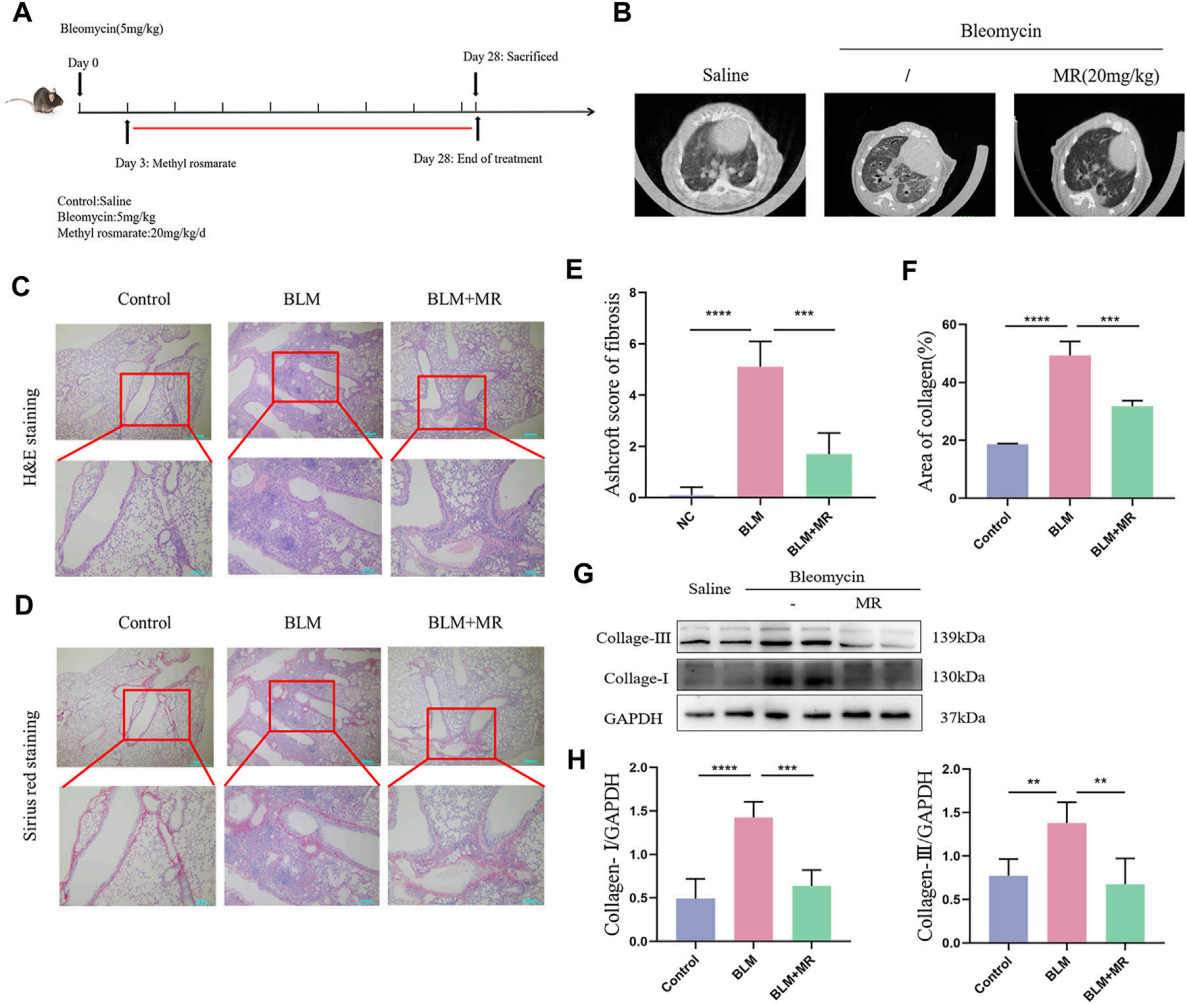
MR attenuates-the BLM-induced pulmonary fibrosis. **(A)** Establishment of a BLM-induced lung fibrosis mouse model. **(B)** MicroCT imaging shows BLM-induced fibrotic changes in both lungs. **(C,D)** H&E and Sirius Red staining to reveal the histopathology of lungs. **(E)** Ashcroft score of fibrosis. **(F)** Statistical analysis of Sirius Red staining. **(G)** Western blot experiments to detect the expression of fibrotic proteins in the lung tissue of MR-treated PF mice. **(H)** Quantitative analysis based on the gray value of each band. Data are expressed as mean ± SD (*p < 0.05, **p < 0.01, and ***p < 0.001).

We investigated the *in vivo* effect of MR on pulmonary fibrosis in a 28-day established model of pulmonary fibrosis in C57BL/6 male mice by tracheal nebulization of BLM. Three days later, we administered the MR treatment daily until the end of day 28 (Figure 3A). Monitoring the body weight changes from day 0–28 and using a small animal MicroCT imaging system revealed that MR significantly improved body weight and reduced fibrosis in both lungs of the treated mice (Figure 3B). H&E and Sirius Red staining showed distortion of the lung structure of the BLM group compared to the untreated controls (Figures 3C,D), with evidence of adhesion of alveolar depressions, and diffuse fibrous proliferation accompanied by the presence of large numbers of inflammatory cell infiltration, and deposition of copious quantities of red-stained collage fibers. By contrast, the BLM mice treated with MR showed significantly improved lung structure and less collagen deposition, which were supported by the Ashcroft score results and statistical data for collagen deposition (Figures 3E,F). Western blot results further confirmed decreases in the levels of fibrosis-associated proteins, such as collagen-Ⅰ and collagen- Ⅲ in the BLM treatment group compared with the BLM group (Figures 3G,H), suggesting that MR attenuated pulmonary fibrosis *in vivo*.

### 3.4 The antifibrotic effects are greater for MR than for RA

**FIGURE 4:**
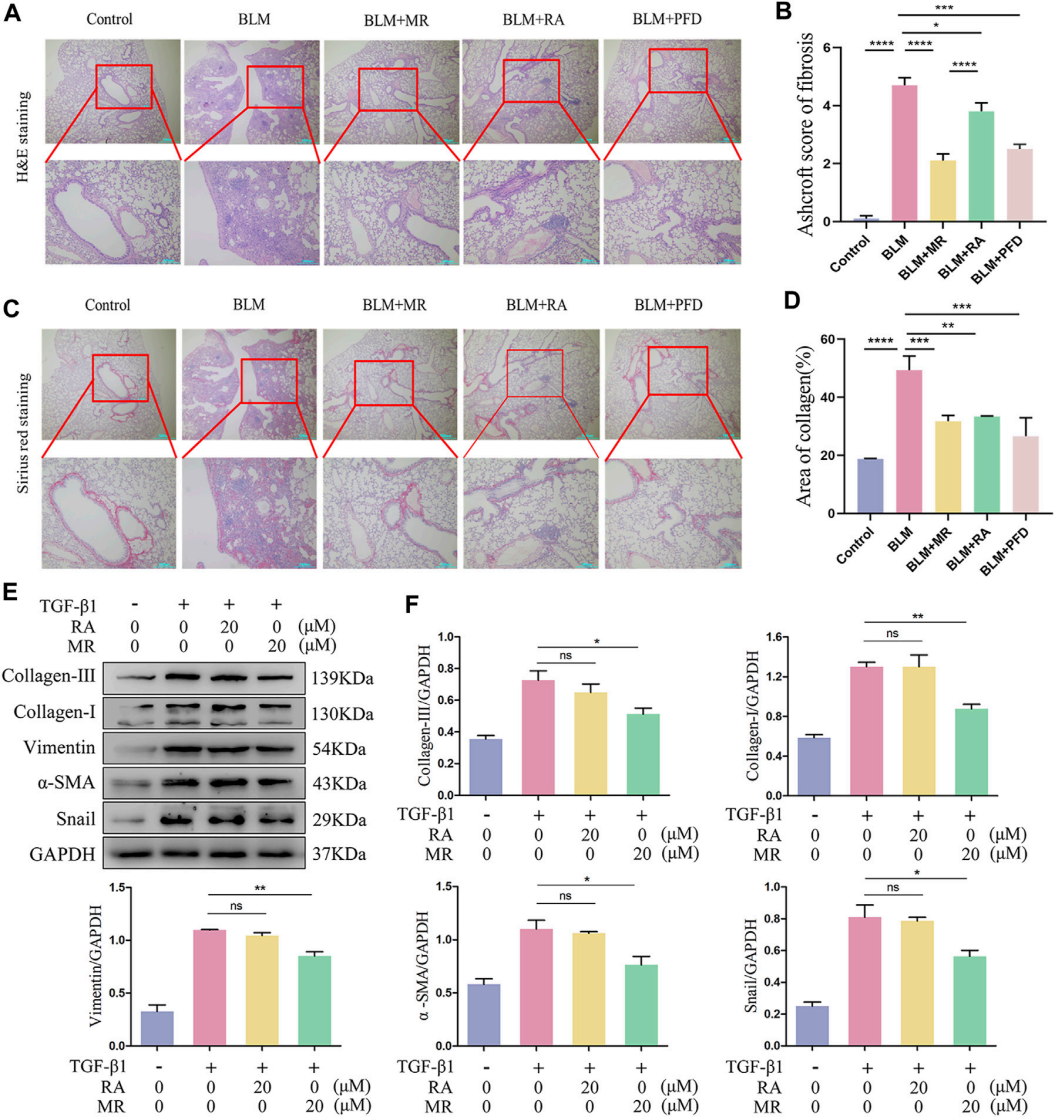
MR has a more potent antifibrotic effect than RA. **(A,C)** H&E and Sirius Red staining to examine the histopathology of lungs. **(B)** Ashcroft score of fibrosis. **(D)** Statistical analysis of Sirius Red staining. **(E)** Analysis of the anti-pulmonary fibrotic effect of MR and RA by Western blots. The expressions of collagen I and III, vimentin, α-SMA, and snail were detected in TGF-β1-stimulated L929 cells. **(F)** Quantitative analysis based on the gray value of each band. Data are expressed as mean ± SD (*p < 0.05, **p < 0.01, and ***p < 0.001).

Currently, the only FDA approved drugs for the treatment of pulmonary fibrosis are pirfenidone and nintedanib (Liu et al., 2017; Sgalla et al., 2018). We used pirfenidone as a positive control, in addition to an RA treatment group, to confirm the better *in vivo* antifibrotic effect of MR. The results of H&E and Sirius Red staining showed significant alteration of the lung tissue morphology of the BLM mice, as indicated by alveolar depressions and adhesions, as well as diffuse fibroplasia accompanied by infiltration of large numbers of inflammatory cells. By contrast, the RA-treated group and the MR-treated group showed a clearer lung tissue texture, narrower alveolar intervals, the presence of fewer inflammatory cells, and a lower degree of lung fibrosis compared with the BLM model group, and the therapeutic effect was better for MR than for RA. The pirfenidone group showed only a small increase in the number of inflammatory cells (Figures 4A,C). The Ashcroft scores and statistical data on collagen deposition also supported a reduction in lung fibrosis (Figures 4B,D). We reasoned that the *in vivo* effect of MR on pulmonary fibrosis was more potent for MR than for the prototype RA drug.

The anti-pulmonary fibrosis effects of MR and RA were also compared and analyzed in our *in vitro* experimental studies on L929 cells treated with 20 μMMR or RA. Western blotting results of the key lung fibrosis proteins revealed significantly greater decreases in the expression of collagen-I, vimentin, α-SMA, and snail proteins in the MR-treated group than in the RA-treated group and untreated controls (Figures 4E,F), suggesting that MR inhibited the expression of key proteins of pulmonary fibrosis, exerting a stronger anti-pulmonary fibrosis effect than was achieved with RA.

### 3.5 MR inhibits TGF-β1-induced L929 cell proliferation and migration

**FIGURE 5:**
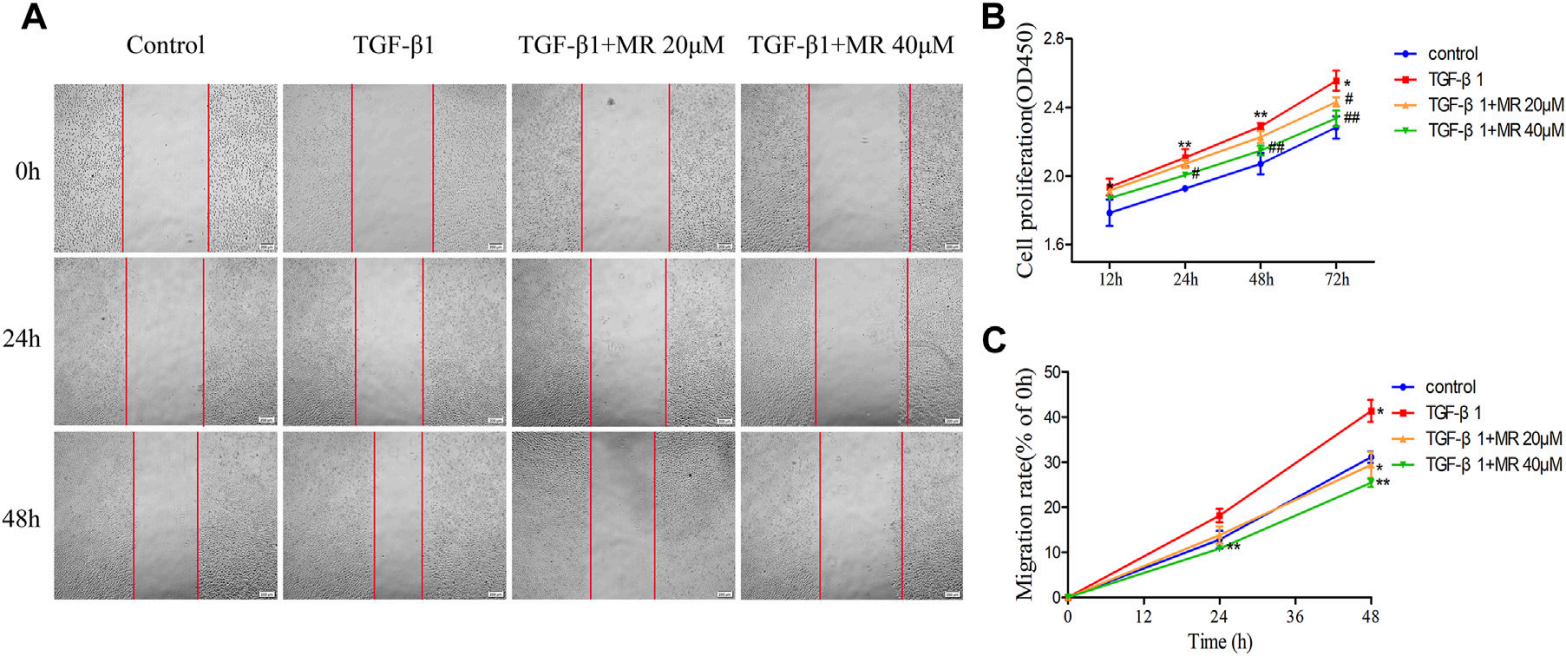
MR inhibits the proliferation and migration of L929 cells induced by TGF-β1. **(A)** Representative wound images of L929 cells treated with MR and TGF-β1. Images were taken at 0, 24, and 48 h after scratching. **(B)** CCK-8 assay to determine the effects of different concentrations of MR on the proliferation of L929 cells induced by TGF-β1. **(C)** Analysis of migrated cells. Data are expressed as mean ± SD (*p < 0.05, **p < 0.01, and ***p < 0.001).

Uncontrolled cell proliferation and high cell migration are pathogenic hallmarks of pulmonary fibrosis. Therefore, we used CCK-8 assay to test the inhibitory effect of MR on TGF-β1-induced proliferation and migration of L929 cells. As shown in Figure 5B, MR, at both doses of 20 μM and 40 μM, can obviously inhibit the proliferation of TGF-β1-induced L929 cells (Figure 5B). The rate of cell migration, tested by scratch assay, was increased by TGF-β1 treatment but decreased by MR co-treatment, indicating that MR inhibited induction of cell migration activity by TGF-β1 (Figures 5A,C).

### 3.6 MR promotes TGF-β1-induced L929 cell apoptosis

**FIGURE 6:**
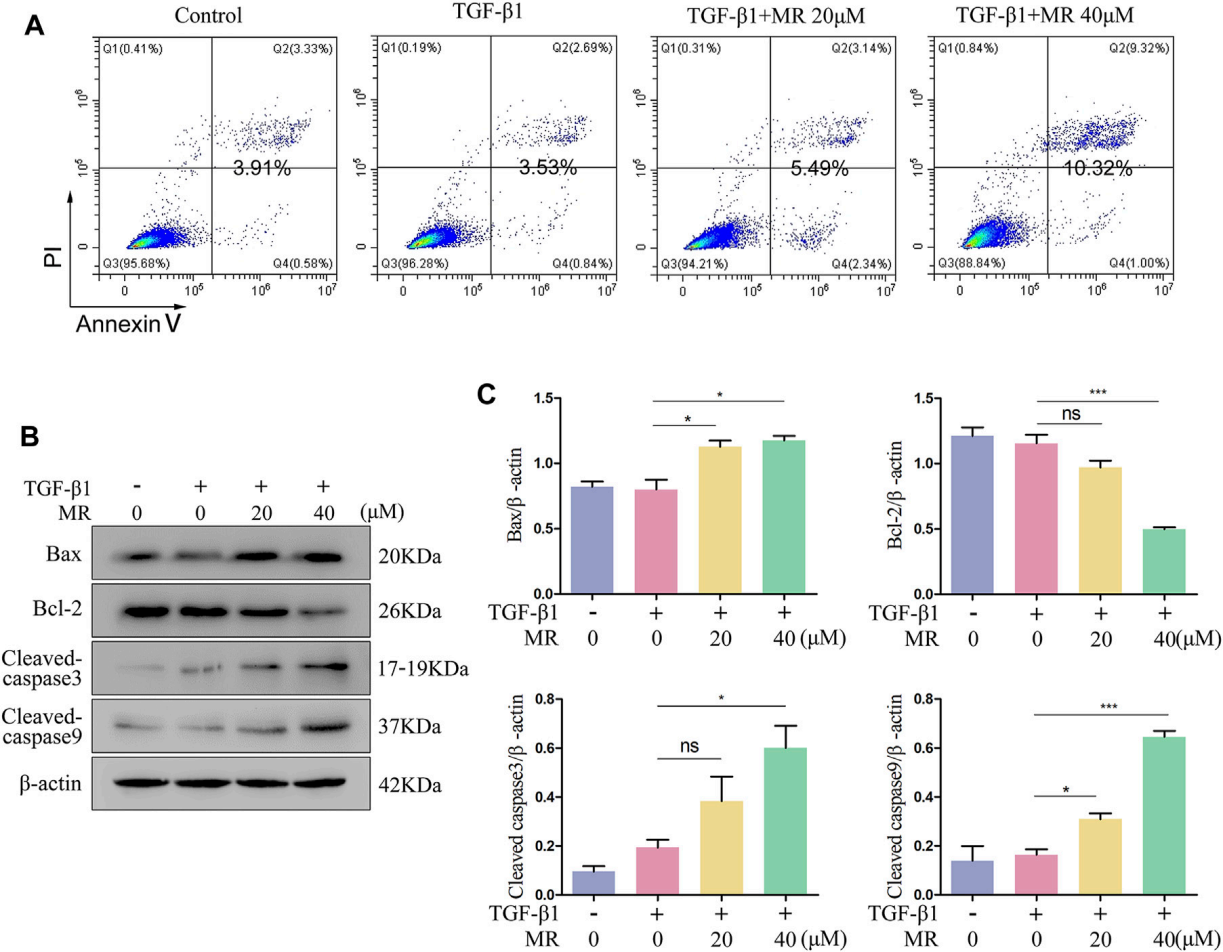
MR promotes the apoptosis of L929 cells in the presence of TGF-β1. **(A)** Detection of the apoptosis rate in MR-treated of L929 cells by flow cytometry. **(B)** Western blot analysis of the expression levels of apoptosis-related proteins in L929 cells 48 h after MR treatment. **(C)** Quantitative analysis based on the gray value of each band. Data are expressed as mean ± SD (*p < 0.05, **p < 0.01, and ***p < 0.001).

As shown in Figure 6A, MR, at both doses of 20 μM and 40 μM, significantly increased the apoptosis of TGF-β1-induced L929 cells (Figure 6A). Examination of the expression of core apoptosis-related proteins revealed significantly greater expression of Bax and cleaved caspase 3 and 9 proteins, and reduced expression of Bcl-2 protein in the MR-treated cells than in the untreated control cells (Figures 6B,C), confirming that MR treatment could promote apoptosis in L929 fibroblasts through the mitochondrial pathway.

### 3.7 MR attenuates fibrotic response by inhibiting the phosphorylation of TGF-β1/smad and MAPK signaling pathways

**FIGURE 7:**
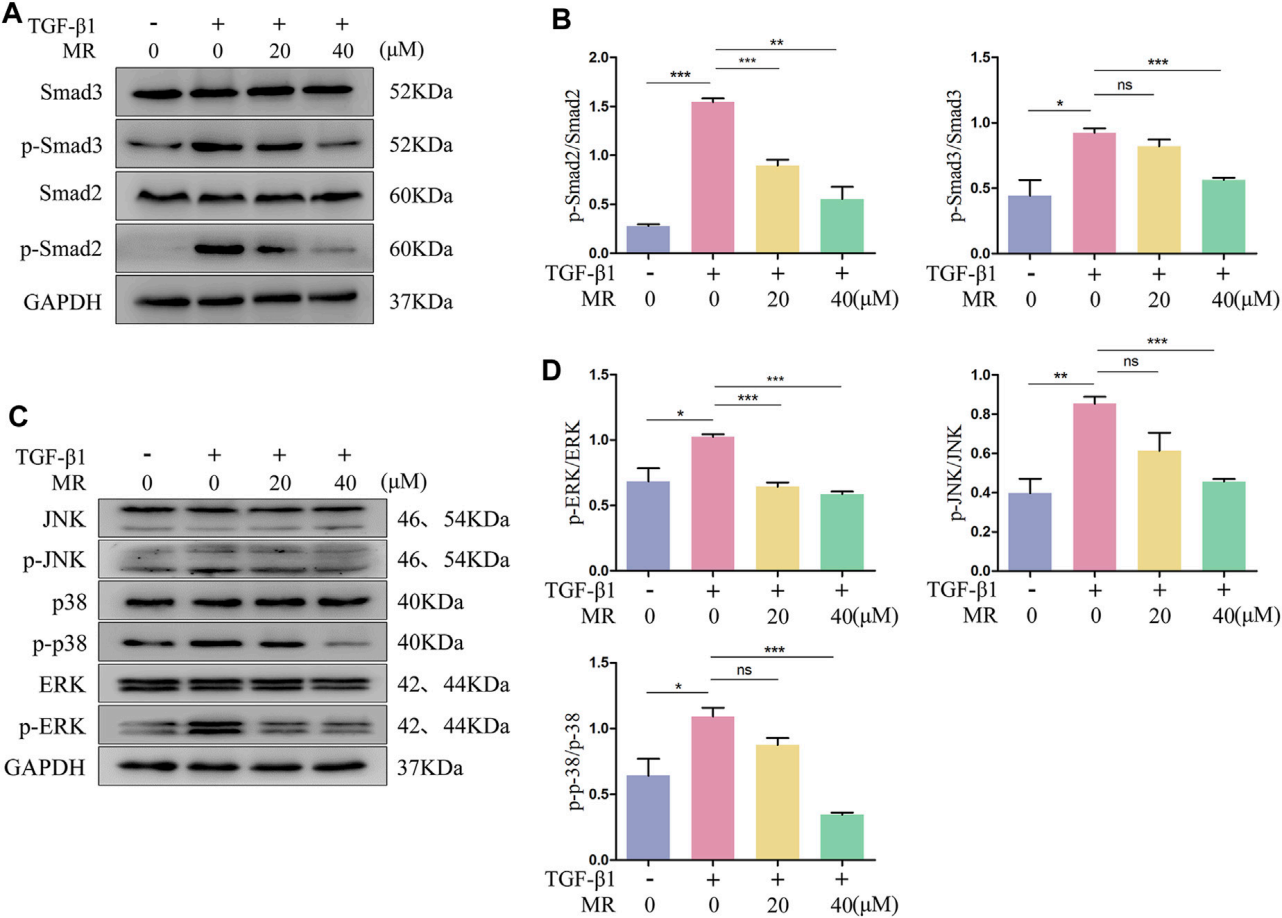
MR attenuates the fibrotic response by inhibiting protein phosphorylation of the TGF-β1/Smad and MAPK signaling pathway in TGF-β1-induced L929 cells. **(A)** Western blot analysis of Smad2, Smad3, p-Smad2, and p-Smad3 expression levels in L929 cells 48 h after MR treatment. **(C)** Western blot analysis of the expression levels of p-p38, p38, p-JNK, JNK, p-ERK, and ERK in L929 cells 48 h after MR treatment. **(B,D)** Quantitative analysis based on the gray value of each band. Data are expressed as mean ± SD (*p < 0.05, **p < 0.01, and ***p < 0.001).

The TGF-β1 signaling pathway significantly affects the regulation of fibroblast activation, in which the key proteins Smad2 and Smad3 are the main downstream regulators that promote TGF-β1-mediated lung fibrosis (Hu et al., 2018). We detected the effect of MR on the key proteins of TGF-β1/Smad signaling pathway by Western blotting, and the results showed that the ratios of p-Smad2/Smad2 and p-Smad3/Smad3 were significantly reduced in TGF-β1-induced L929 cells treated with MR for 48 h (Luo et al., 2021; Li Y. et al., 2022; Xiao et al., 2023) (Figures 7A,B). TGF-β1 treatment alone also showed elevated p-p38/p38, p-JNK/JNK, and p-ERK/ERK expression ratios and significant reductions in these ratios after the addition of MR (Figures 7C,D). These data suggested that MR may exert its antifibrotic effects by inhibiting the activation of the TGF-β1/Smad and MAPK signaling pathways.

## 4 Discussion

In the present study, an industrially scalable MR process using Salvia castanea Diels f. tomentosa Stib. as the starting material has been developed, which includes steps such as polyamide resin chromatography, crystallization and esterification. *Salvia castanea* Diels f. *tomentosa* Stib., a variant of Salvia castanea, is a unique species in Xizang, China and mainly grows in Nyingchi district of Xizang, with a storage capacity of over 5000ts (Ye et al., 2004), and can be sustainable development and utilization through artificial cultivation. *Salvia castanea* Diels f. *tomentosa* Stib. is used locally in Tibet as a substitute for *S. miltiorrhiza* Bge. However, its main phenolic acid component is RA, not salvianolic acid B. Due to the catalytic effect of hydrochloric acid, RA can react with methanol to generate MR, therefore, *S. castanea* Diels f. *tomentosa* Stib. is a valuable and potential source of MR.

Previous studies have shown that MR has multiple biological effects, such as anti-inflammatory, antioxidant, and anti-tumor. For example, MR can inhibit the LPS-induced expression of pro-inflammatory cytokines including IL-1β and IL-6, and the production of NO via suppression of the MyD88-NF-κB signaling pathway (So et al., 2016). MR can induce autophagy and apoptosis in cervical cancer cells by inhibiting mTOR-S6K1 signaling (Nam et al., 2019), also can effectively inhibit ovarian cancer cell migration and reverse cisplatin resistance by inhibiting the expression of FOXM1 (Lim et al., 2020). MR with stronger activity and easier oral absorption, making it more suitable for development as an anti-pulmonary fibrosis drug than RA. In the present study, we first investigated the antifibrotic effect of MR using a mouse model of pulmonary fibrosis and found that, at the doses used in these experiments, the anti-fibrotic effects of MR were slightly inferior to those of pirfenidone, which indicates that MR has high scientific research value, and remains a promising clinical prospect for treating lung fibrosis due to its unique advantages, including its abundant natural sources.

The process of fibrosis is extremely complex and can involve changes in a variety of components associated with lung function. Myofibroblasts are the key effector cells responsible for the initiation and development of pulmonary fibrosis. Upon transformation from fibroblasts, myofibroblasts express increased levels of α-SMA, vimentin, and snail, and promote collage-I and collagen-III deposition (Li et al., 2018). In pulmonary fibrosis, snail is involved in the TGF-β1-induced endothelial−mesenchymal transition (Hashimoto et al., 2010), the promotion of epithelial cell apoptosis and the inhibition of fibroblast and myofibroblast apoptosis (Mo et al., 2015). Collagen I and III, which are the major water-insoluble fibrous proteins in the body, are present in the ECM, and their excessive deposition and abnormal degradation impede injury healing and lead to fibrosis (Devos et al., 2023; Xu et al., 2023). In this report, we used collagen-Ⅰ, collagen-Ⅲ, α-SMA, vimentin, and snail as biochemical indicators to evaluate the degree of pulmonary fibrosis. Our western blotting results confirmed that expression of all these biochemical indicators increased in our mouse model, and MR treatment resulted in dose-dependent reversal of these increases. MMP-1, also known as collagenase 1 or fibroblast collagenase, can degrade type I-III collagens *in vitro* (Chuliá-Peris et al., 2022). One previous study detected that MR could inhibit MMP-1, using a fluorometric assay on purified enzymes *in vitro*, which indicates that MR may inhibit degradation of collagen I and collagen III *in vivo* (Yuan et al., 2013). However, collagen production and degradation are two distinct processes, and although *in vitro* experiments have shown that MR inhibits MMP-1 activity, our experimental results indicate that the ability of MR to reduce collagen synthesis is much greater than its ability to inhibit MMP-1-mediated collagen degradation. In addition, matrix stiffening synergizes with TGF-β1 can promote mRNA expression of both col1a1 and mmp1 in IPF fibroblasts, and highly expressed MMP1 mainly presents in the vicinity of alveolar epithelial cells rather than in the interstitial space where collagen is deposited (Chuliá-Peris et al., 2022). The exact role of MMP-1, as well as the inhibition effect of MR on MMP-1 activity in pulmonary fibrosis need further investigation.

Our further studies confirmed that MR could significantly inhibit the proliferation and migration of L929 cells induced by TGF- β1 and induce its apoptosis as well. The expression of pro-apoptotic protein bax, cleaved-caspase 9 and cleaved-caspase 3 increased, while the expression of anti-apoptotic protein Bcl-2 was inhibited, suggesting that MR may promote apoptosis of mitochondrial pathway by regulating the expression of apoptosis-related proteins. Interference in or inhibition of apoptosis and the resulting persistence of myofibroblasts are well documented to promote the formation of fibroblast foci and the secretion of large amounts of ECM, leading to the progressive development of pulmonary fibrosis (Phan, 2002). Above results confirmed that MR had anti-fibrotic effect *in vitro*.

Several previous sudies have confirmed TGF-β1 as a key mediator of fibrotic changes and that TGF-β1/Smad signaling is the main signaling pathway leading to pulmonary fibrosis (Djudjaj and Boor, 2019). In this report, we showed significant decreases in the phosphorylation ratio of Smad2 and Smad3 in response to MR treatment, suggesting that MR inhibited the activation of the TGF-β1/Smad pathway to produce its antifibrotic effect in the lung. MAPK signaling pathway, is one important non-Smad pathways mediated by TGF-β1 in the development of pulmonary fibrosis. Several studies indicate that TGF-β1 markedly enhance MAPK signaling and cause human lung fibroblasts-myofibroblasts transformation and epithelial-mesenchymal transition (EMT) (Li et al., 2023). Here, we proved that MR significantly decreased the phosphorylation levels of JNK, p-38, and ERK, which indicates that MR alleviates pulmonary fibrosis also by inhibition of the MAPK pathway.

In conclusion, we developed a scalable MR preparation process using Salvia castanea Diels f. tomentosa Stib. as the starting material and demonstrated that MR has potent antifibrotic activity *in vitro* and *in vivo*. In addition, several previous studies investigated the safety of MR and some compounds in which MR is one main active ingredient, and no obvious toxicity has been found *in vitro* and *in vivo* (Lim et al., 2014; Flores-Bocanegra et al., 2017; Giles-Rivas et al., 2020). Therefore, MR is a candidate therapeutic compound for pulmonary fibrosis patients, and establishment of this scalable MR preparation technology increases its feasibility. We hope that more research will be conducted in this field in the future to bring new therapeutic options for patients with pulmonary fibrosis.

## Data Availability

The original contributions presented in the study are included in the article/Supplementary Material, further inquiries can be directed to the corresponding authors.

## References

[B1] Andugulapati S. B. Gourishetti K. Tirunavalli S. K. Shaikh T. B. Sistla R. (2020). Biochanin-A ameliorates pulmonary fibrosis by suppressing the TGF-β mediated EMT, myofibroblasts differentiation and collagen deposition in *in vitro* and *in vivo* systems. Phytomedicine 78, 153298. 10.1016/j.phymed.2020.153298 32781391 PMC7395646

[B2] Bahri S. Ali R. B. Abdennabi R. Nahdi A. Mlika M. Jameleddine S. (2021). Industrial elimination of essential oils from rosmarinus officinalis: in support of the synergic antifibrotic effect of rosmarinic and carnosic acids in bleomycin model of lung fibrosis. Nutr. Cancer. 73 (11-12), 2376–2387. 10.1080/01635581.2020.1826991 33059466

[B3] Bahri S. Mies F. Ben A. R. Mlika M. Jameleddine S. Mc E. K. (2017). Rosmarinic acid potentiates carnosic acid induced apoptosis in lung fibroblasts. PLoS One 12 (9), e0184368. 10.1371/journal.pone.0184368 28877257 PMC5587316

[B4] Cai G. Lin F. Wu D. Lin C. Chen H. Wei Y. (2022). Rosmarinic acid inhibits mitochondrial damage by alleviating unfolded protein response. Front. Pharmacol. 13, 859978. 10.3389/fphar.2022.859978 35652041 PMC9149082

[B5] Camelo A. Dunmore R. Sleeman M. A. Clarke D. L. (2014). The epithelium in idiopathic pulmonary fibrosis: breaking the barrier. Front. Pharmacol. 4, 173. 10.3389/fphar.2013.00173 24454287 PMC3887273

[B6] Chu X. Ci X. He J. Jiang L. Wei M. Cao Q. (2012). Effects of a natural prolyl oligopeptidase inhibitor, rosmarinic acid, on lipopolysaccharide-induced acute lung injury in mice. Molecules 17 (3), 3586–3598. 10.3390/molecules17033586 22441336 PMC6269028

[B7] Chuliá-Peris L. Carreres-Rey C. Gabasa M. Alcaraz J. Carretero J. Pereda J. (2022). Matrix metalloproteinases and their inhibitors in pulmonary fibrosis: EMMPRIN/CD147 comes into play. Int. J. Mol. Sci. 23 (13), 6894. 10.3390/ijms23136894 35805895 PMC9267107

[B8] Dempsey O. J. (2006). Clinical review: idiopathic pulmonary fibrosis--past, present and future. Respir. Med. 100 (11), 1871–1885. 10.1016/j.rmed.2006.08.017 16987645

[B9] Devos H. Zoidakis J. Roubelakis M. G. Latosinska A. Vlahou A. (2023). Reviewing the regulators of COL1A1. Int. J. Mol. Sci. 24 (12), 10004. 10.3390/ijms241210004 37373151 PMC10298483

[B10] Djudjaj S. Boor P. (2019). Cellular and molecular mechanisms of kidney fibrosis. Mol. Asp. Med. 65, 16–36. 10.1016/j.mam.2018.06.002 29909119

[B11] Dubois M. Bailly F. Mbemba G. Mouscadet J. F. Debyser Z. Witvrouw M. (2008). Reaction of rosmarinic acid with nitrite ions in acidic conditions: discovery of nitro- and dinitrorosmarinic acids as new anti-HIV-1 agents. J. Med. Chem. 51 (8), 2575–2579. 10.1021/jm7011134 18351727

[B12] Flores-Bocanegra L. González-Andrade M. Bye R. Linares E. Mata R. (2017). α-Glucosidase inhibitors from Salvia circinata. J. Nat. Prod. 80 (5), 1584–1593. 10.1021/acs.jnatprod.7b00155 28422509

[B13] George P. M. Patterson C. M. Reed A. K. Thillai M. (2019). Lung transplantation for idiopathic pulmonary fibrosis. Lancet Resp. Med. 7 (3), 271–282. 10.1016/S2213-2600(18)30502-2 30738856

[B14] Giles-Rivas D. Estrada-Soto S. Aguilar-Guadarrama A. B. Almanza-Pérez J. García-Jiménez S. Colín-Lozano B. (2020). Antidiabetic effect of Cordia morelosana, chemical and pharmacological studies. J. Ethnopharmacol. 251, 112543. 10.1016/j.jep.2020.112543 31917279

[B15] Gul I. Hassan A. Haq E. Ahmad S. M. Shah R. A. Ganai N. A. (2023). An investigation of the antiviral potential of phytocompounds against avian infectious bronchitis virus through template-based molecular docking and molecular dynamics simulation analysis. Viruses 15 (4), 847. 10.3390/v15040847 37112828 PMC10144825

[B16] Hashimoto N. Phan S. H. Imaizumi K. Matsuo M. Nakashima H. Kawabe T. (2010). Endothelial-mesenchymal transition in bleomycin-induced pulmonary fibrosis. Am. J. Respir. Cell. Mol. Biol. 43 (2), 161–172. 10.1165/rcmb.2009-0031OC 19767450 PMC2937229

[B17] Hu H. H. Chen D. Q. Wang Y. N. Feng Y. L. Cao G. Vaziri N. D. (2018). New insights into TGF-β/Smad signaling in tissue fibrosis. Chem. Biol. Interact. 292, 76–83. 10.1016/j.cbi.2018.07.008 30017632

[B18] Huh J. Y. Lee J. H. Song J. W. (2023). Efficacy and safety of combination therapy with pirfenidone and nintedanib in patients with idiopathic pulmonary fibrosis. Front. Pharmacol. 14, 1301923. 10.3389/fphar.2023.1301923 38192410 PMC10773730

[B19] Kim D. S. Kim H. R. Woo E. R. Hong S. T. Chae H. J. Chae S. W. (2005). Inhibitory effects of rosmarinic acid on adriamycin-induced apoptosis in H9c2 cardiac muscle cells by inhibiting reactive oxygen species and the activations of c-Jun N-terminal kinase and extracellular signal-regulated kinase. Biochem. Pharmacol. 70 (7), 1066–1078. 10.1016/j.bcp.2005.06.026 16102732

[B20] King T. J. Bradford W. Z. Castro-Bernardini S. Fagan E. A. Glaspole I. Glassberg M. K. (2014). A phase 3 trial of pirfenidone in patients with idiopathic pulmonary fibrosis. N. Engl. J. Med. 370 (22), 2083–2092. 10.1056/NEJMoa1402582 24836312

[B21] Koudstaal T. Funke-Chambour M. Kreuter M. Molyneaux P. L. Wijsenbeek M. S. (2023). Pulmonary fibrosis: from pathogenesis to clinical decision-making. Trends Mol. Med. 29 (12), 1076–1087. 10.1016/j.molmed.2023.08.010 37716906

[B22] Ley B. Collard H. R. (2013). Epidemiology of idiopathic pulmonary fibrosis. Clin. Epidemiol. 5, 483–492. 10.2147/CLEP.S54815 24348069 PMC3848422

[B23] Li J. Wei Q. Song K. Wang Y. Yang Y. Li M. (2023). Tangeretin attenuates bleomycin-induced pulmonary fibrosis by inhibiting epithelial-mesenchymal transition via the PI3K/Akt pathway. Front. Pharmacol. 14, 1247800. 10.3389/fphar.2023.1247800 37781713 PMC10540689

[B24] Li T. Chen Y. Li Y. Chen G. Zhao Y. Su G. (2022a). Antifibrotic effect of AD-1 on lipopolysaccharide-mediated fibroblast injury in L929 cells and bleomycin-induced pulmonary fibrosis in mice. Food Funct. 13 (14), 7650–7665. 10.1039/d1fo04212b 35735105

[B25] Li X. H. Xiao T. Yang J. H. Qin Y. Gao J. J. Liu H. J. (2018). Parthenolide attenuated bleomycin-induced pulmonary fibrosis via the NF-κB/Snail signaling pathway. Respir. Res. 19 (1), 111. 10.1186/s12931-018-0806-z 29871641 PMC5989384

[B26] Li Y. Wang L. Zhang Q. Tian L. Gan C. Liu H. (2022b). Blueberry juice attenuates pulmonary fibrosis via blocking the TGF-β1/smad signaling pathway. Front. Pharmacol. 13, 825915. 10.3389/fphar.2022.825915 35418869 PMC8996108

[B27] Liang Z. Xu Y. Wen X. Nie H. Hu T. Yang X. (2016). Rosmarinic acid attenuates airway inflammation and hyperresponsiveness in a murine model of asthma. Molecules 21 (6), 769. 10.3390/molecules21060769 27304950 PMC6274450

[B28] Lim H. J. Woo K. W. Lee K. R. Lee S. K. Kim H. P. (2014). Inhibition of proinflammatory cytokine generation in lung inflammation by the leaves of perilla frutescens and its constituents. Biomol. Ther. 22 (1), 62–67. 10.4062/biomolther.2013.088 PMC3936423 24596623

[B29] Lim S. H. Nam K. H. Kim K. Yi S. A. Lee J. Han J. W. (2020). Rosmarinic acid methyl ester regulates ovarian cancer cell migration and reverses cisplatin resistance by inhibiting the expression of forkhead box M1. Pharmaceuticals 13 (10), 302. 10.3390/ph13100302 33053721 PMC7601071

[B30] Lin L. Zhu D. Zou L. Yang B. Zhao M. (2013). Antibacterial activity-guided purification and identification of a novel C-20 oxygenated ent-kaurane from Rabdosia serra (MAXIM.) HARA. Food Chem. 139 (1-4), 902–909. 10.1016/j.foodchem.2013.01.001 23561188

[B31] Liu Y. M. Nepali K. Liou J. P. (2017). Idiopathic pulmonary fibrosis: current status, recent progress, and emerging targets. J. Med. Chem. 60 (2), 527–553. 10.1021/acs.jmedchem.6b00935 28122457

[B32] Luo X. Deng Q. Xue Y. Zhang T. Wu Z. Peng H. (2021). Anti-fibrosis effects of magnesium lithospermate B in experimental pulmonary fibrosis: by inhibiting TGF-βri/smad signaling. Molecules 26 (6), 1715. 10.3390/molecules26061715 33808650 PMC8003516

[B33] Mei Q. Liu Z. Zuo H. Yang Z. Qu J. (2021). Idiopathic pulmonary fibrosis: an update on pathogenesis. Front. Pharmacol. 12, 797292. 10.3389/fphar.2021.797292 35126134 PMC8807692

[B34] Mo X. T. Zhou W. C. Cui W. H. Li D. L. Li L. C. Xu L. (2015). Inositol-requiring protein 1 - X-box-binding protein 1 pathway promotes epithelial-mesenchymal transition via mediating snail expression in pulmonary fibrosis. Int. J. Biochem. Cell Biol. 65, 230–238. 10.1016/j.biocel.2015.06.006 26065400

[B35] Nam K. H. Yi S. A. Nam G. Noh J. S. Park J. W. Lee M. G. (2019). Identification of a novel S6K1 inhibitor, rosmarinic acid methyl ester, for treating cisplatin-resistant cervical cancer. BMC Cancer 19 (1), 773. 10.1186/s12885-019-5997-2 31387554 PMC6683399

[B36] Osakabe N. Yasuda A. Natsume M. Sanbongi C. Kato Y. Osawa T. (2002). Rosmarinic acid, a major polyphenolic component of Perilla frutescens, reduces lipopolysaccharide (LPS)-induced liver injury in D-galactosamine (D-GalN)-sensitized mice. Free. Radic. Biol. Med. 33 (6), 798–806. 10.1016/s0891-5849(02)00970-x 12208367

[B37] Phan S. H. (2002). The myofibroblast in pulmonary fibrosis. Chest 122 (6), 286S–289S. 10.1378/chest.122.6_suppl.286s 12475801

[B38] Raghu G. Collard H. R. Egan J. J. Martinez F. J. Behr J. Brown K. K. (2011). An official ATS/ERS/JRS/ALAT statement: idiopathic pulmonary fibrosis: evidence-based guidelines for diagnosis and management. Am. J. Respir. Crit. Care Med. 183 (6), 788–824. 10.1164/rccm.2009-040GL 21471066 PMC5450933

[B39] Richeldi L. du Bois R. M. Raghu G. Azuma A. Brown K. K. Costabel U. (2014). Efficacy and safety of nintedanib in idiopathic pulmonary fibrosis. N. Engl. J. Med. 370 (22), 2071–2082. 10.1056/NEJMoa1402584 24836310

[B40] Saiko P. Steinmann M. T. Schuster H. Graser G. Bressler S. Giessrigl B. (2015). Epigallocatechin gallate, ellagic acid, and rosmarinic acid perturb dNTP pools and inhibit *de novo* DNA synthesis and proliferation of human HL-60 promyelocytic leukemia cells: synergism with arabinofuranosylcytosine. Phytomedicine 22 (1), 213–222. 10.1016/j.phymed.2014.11.017 25636891

[B41] Schniering J. Guo L. Brunner M. Schibli R. Ye S. Distler O. (2018). Evaluation of (99m)Tc-rhAnnexin V-128 SPECT/CT as a diagnostic tool for early stages of interstitial lung disease associated with systemic sclerosis. Arthritis Res. Ther. 20 (1), 183. 10.1186/s13075-018-1681-1 30115119 PMC6097327

[B42] Sgalla G. Iovene B. Calvello M. Ori M. Varone F. Richeldi L. (2018). Idiopathic pulmonary fibrosis: pathogenesis and management. Respir. Res. 19 (1), 32. 10.1186/s12931-018-0730-2 29471816 PMC5824456

[B43] Slobodníková L. Fialová S. Hupková H. Grancai D. (2013). Rosmarinic acid interaction with planktonic and biofilm *Staphylococcus aureus* . Nat. Prod. Commun. 8 (12), 1747–1750. 10.1177/1934578x1300801223 24555289

[B44] So Y. Lee S. Y. Han A. R. Kim J. B. Jeong H. G. Jin C. H. (2016). Rosmarinic acid methyl ester inhibits LPS-induced NO production via suppression of MyD88- dependent and -independent pathways and induction of HO-1 in RAW 264.7 cells. Molecules 21 (8), 1083. 10.3390/molecules21081083 27548124 PMC6274143

[B45] Wang S. Yu J. Liu Y. Yu J. Ma Y. Zhou L. (2024). Bletilla striata polysaccharide attenuated the progression of pulmonary fibrosis by inhibiting TGF-β1/Smad signaling pathway. J. Ethnopharmacol. 323, 117680. 10.1016/j.jep.2023.117680 38171465

[B46] Xiao T. Gao D. Gu X. Zhang Y. Zhu Y. Zhang Z. (2023). Flavokawain A ameliorates pulmonary fibrosis by inhibiting the TGF-β signaling pathway and CXCL12/CXCR4 axis. Eur. J. Pharmacol. 958, 175981. 10.1016/j.ejphar.2023.175981 37579968

[B47] Xu M. Zhao C. Song H. Wang C. Li H. Qiu X. (2023). Inhibitory effects of Schisandrin C on collagen behavior in pulmonary fibrosis. Sci. Rep. 13 (1), 13475. 10.1038/s41598-023-40631-6 37596361 PMC10439186

[bib52] Ye H. Liao W. Li H. (2004). Resource investigation of “Linzhi Danshen”. Traditional Chinese Medicine 11, 809–811. 10.13863/j.issn1001-4454.2004.11.008

[B48] Yuan H. Lu W. Wang L. Shan L. Li H. Huang J. (2013). Synthesis of derivatives of methyl rosmarinate and their inhibitory activities against matrix metalloproteinase-1 (MMP-1). Eur. J. Med. Chem. 62, 148–157. 10.1016/j.ejmech.2012.09.047 23353736

[B49] Zhang J. Chen X. Chen H. Li R. Xu P. Lv C. (2020a). Engeletin ameliorates pulmonary fibrosis through endoplasmic reticulum stress depending on lnc949-mediated TGF-β1-Smad2/3 and JNK signalling pathways. Pharm. Biol. 58 (1), 1105–1114. 10.1080/13880209.2020.1834590 33181025 PMC7671710

[B50] Zhang T. Ma S. Liu C. Hu K. Xu M. Wang R. (2020b). Rosmarinic acid prevents radiation-induced pulmonary fibrosis through attenuation of ROS/MYPT1/TGFβ1 signaling via miR-19b-3p. Dose-Response 18 (4), 1559325820968413. 10.1177/1559325820968413 33149731 PMC7580151

[B51] Zhang Y. Liu Q. Ning J. Jiang T. Kang A. Li L. (2022). The proteasome-dependent degradation of ALKBH5 regulates ECM deposition in PM(2.5) exposure-induced pulmonary fibrosis of mice. J. Hazard. Mat. 432, 128655. 10.1016/j.jhazmat.2022.128655 35334267

