# Supplementary figures and images for: Anti-pulmonary fibrosis activity analysis of methyl rosmarinate obtained from Salvia castanea Diels f. tomentosa Stib. using a scalable process
Source: Front Pharmacol. 2024 Jun 4;15:1374669. doi: 10.3389/fphar.2024.1374669 (PMC11183283; doi:10.3389/fphar.2024.1374669)

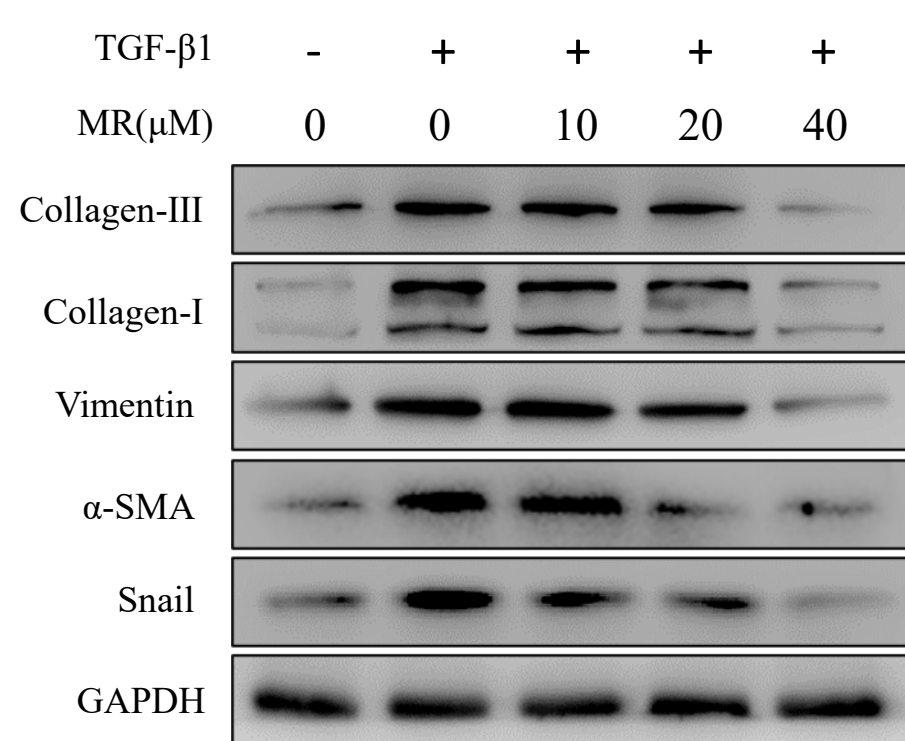

Figure 1B

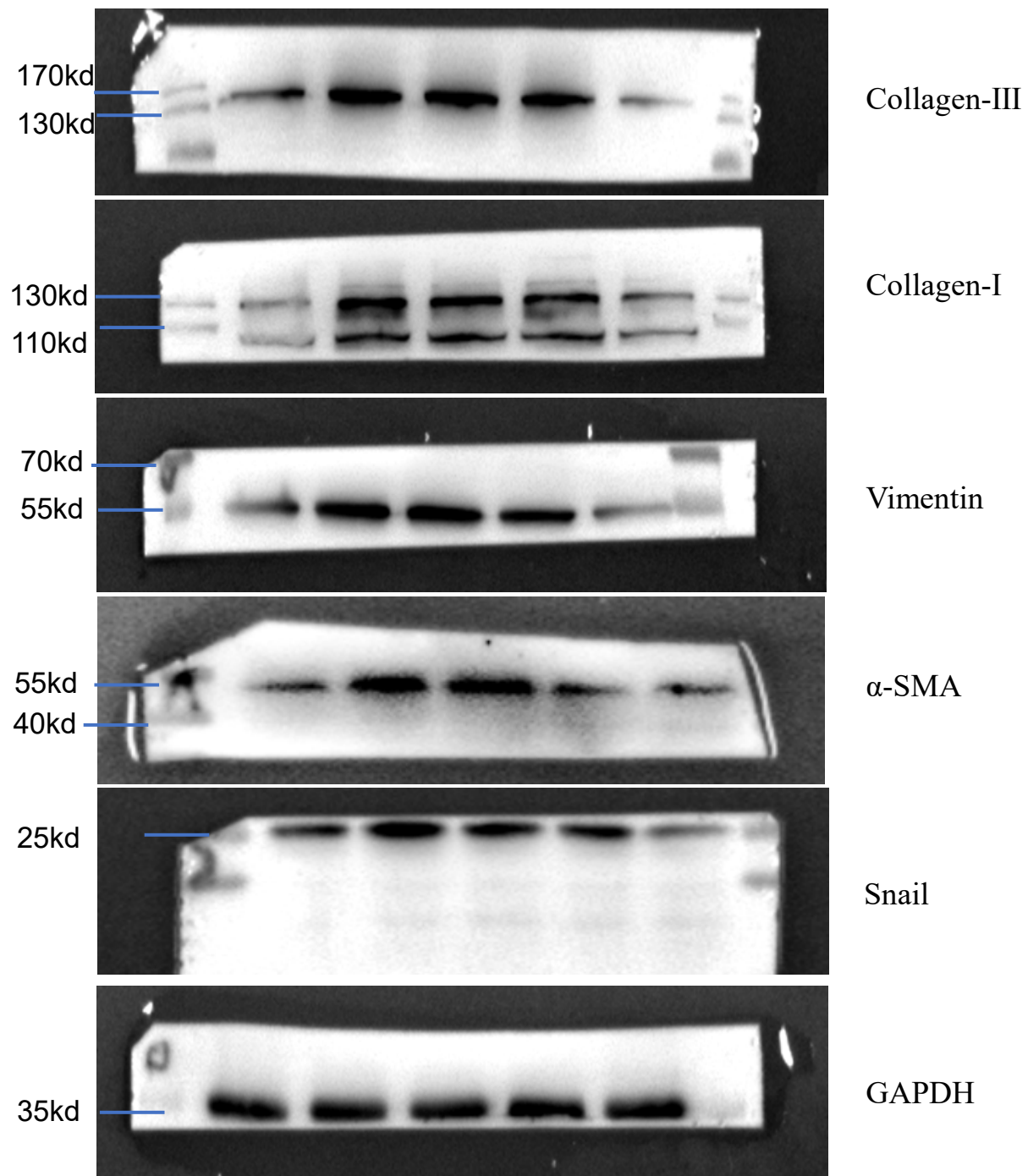

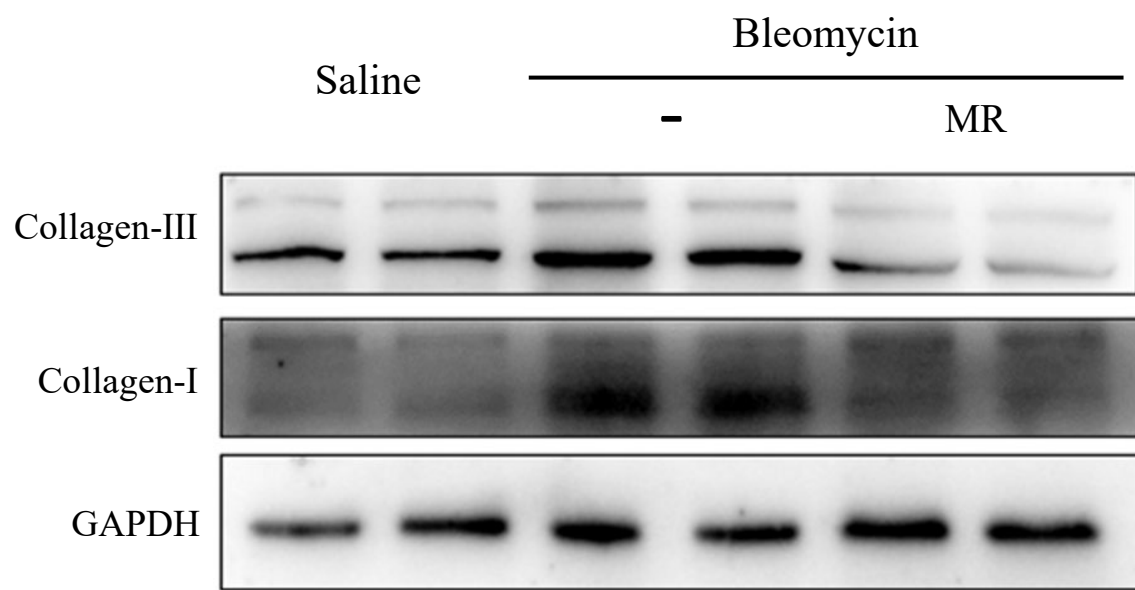

Figure 2G

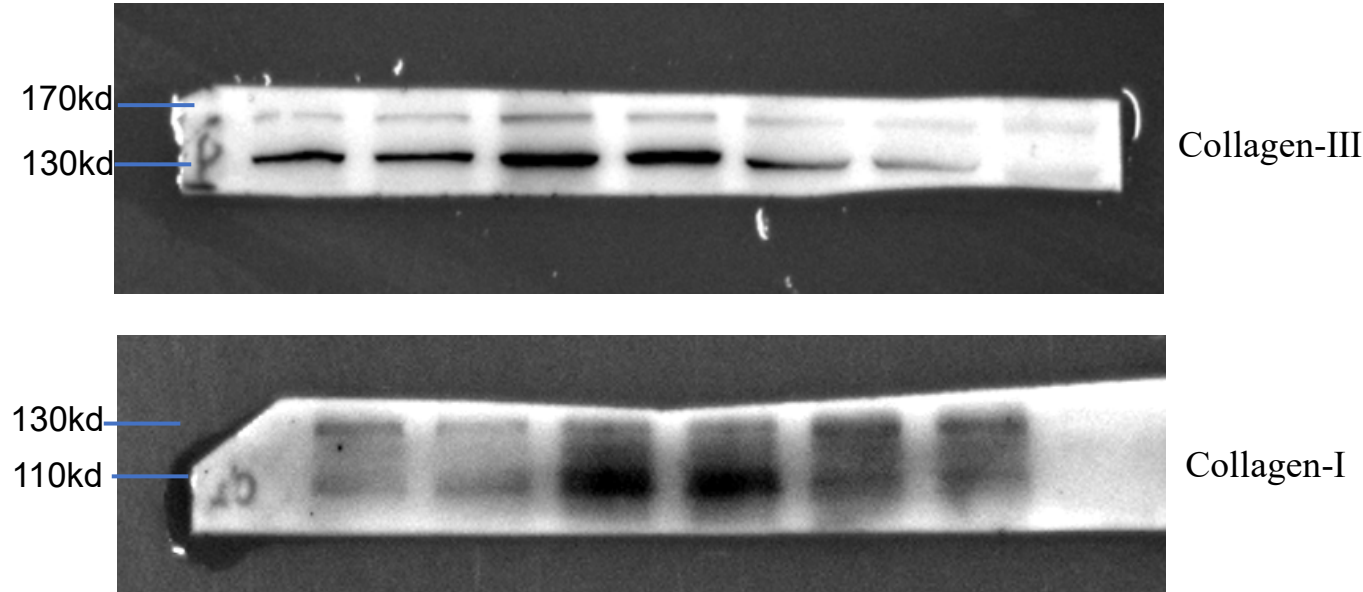

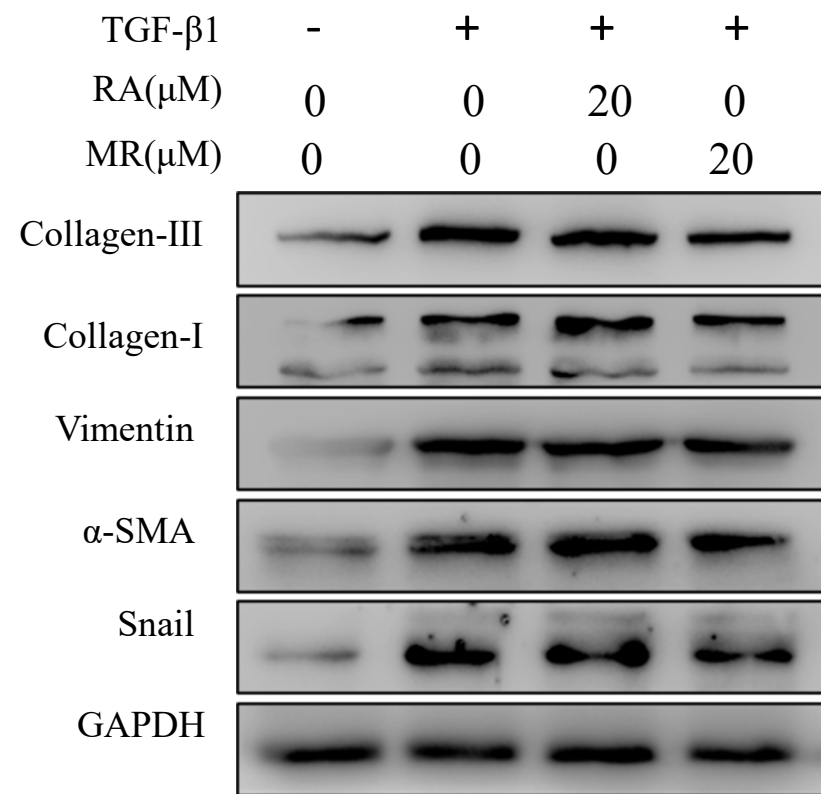

Figure 3E

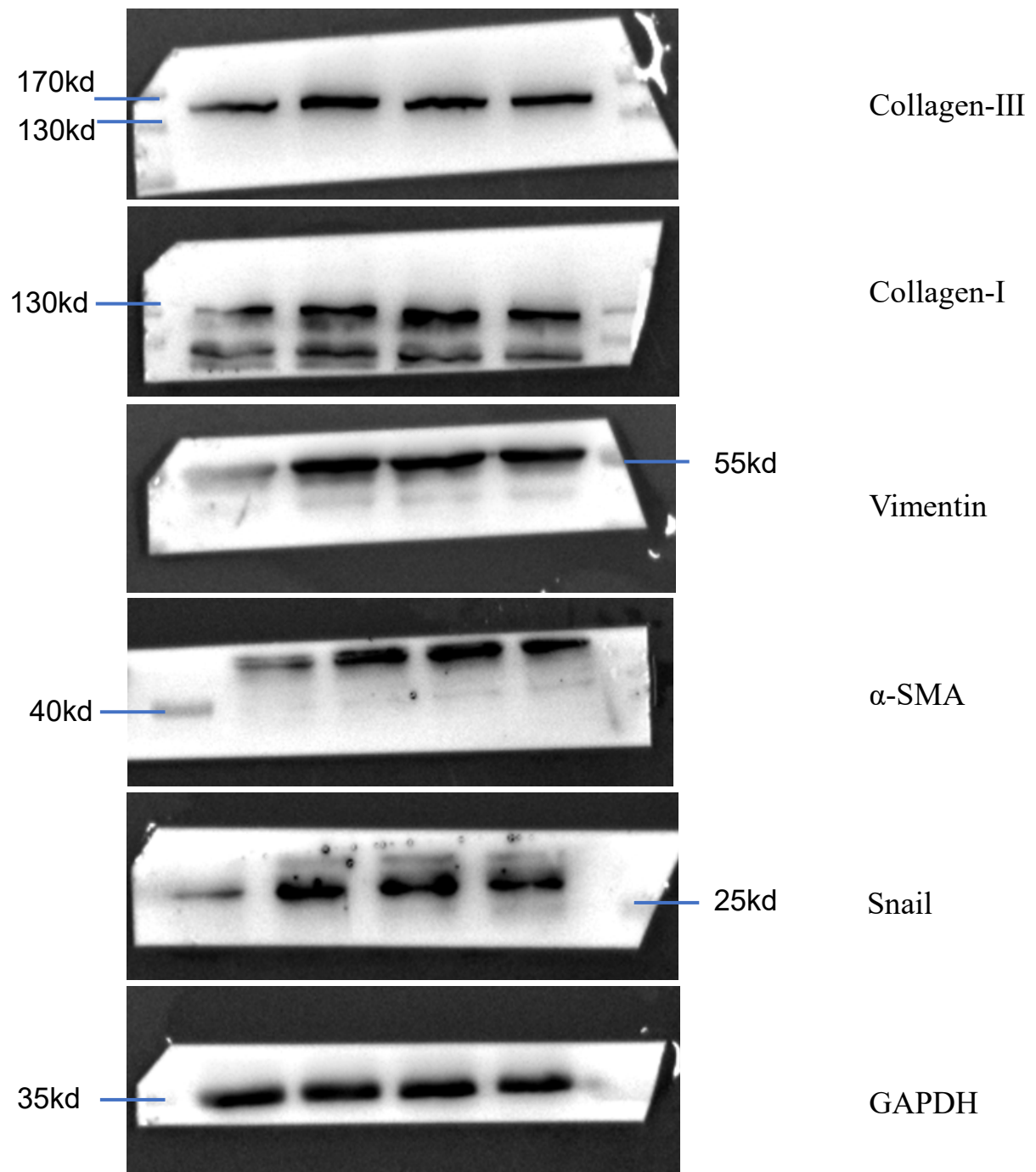

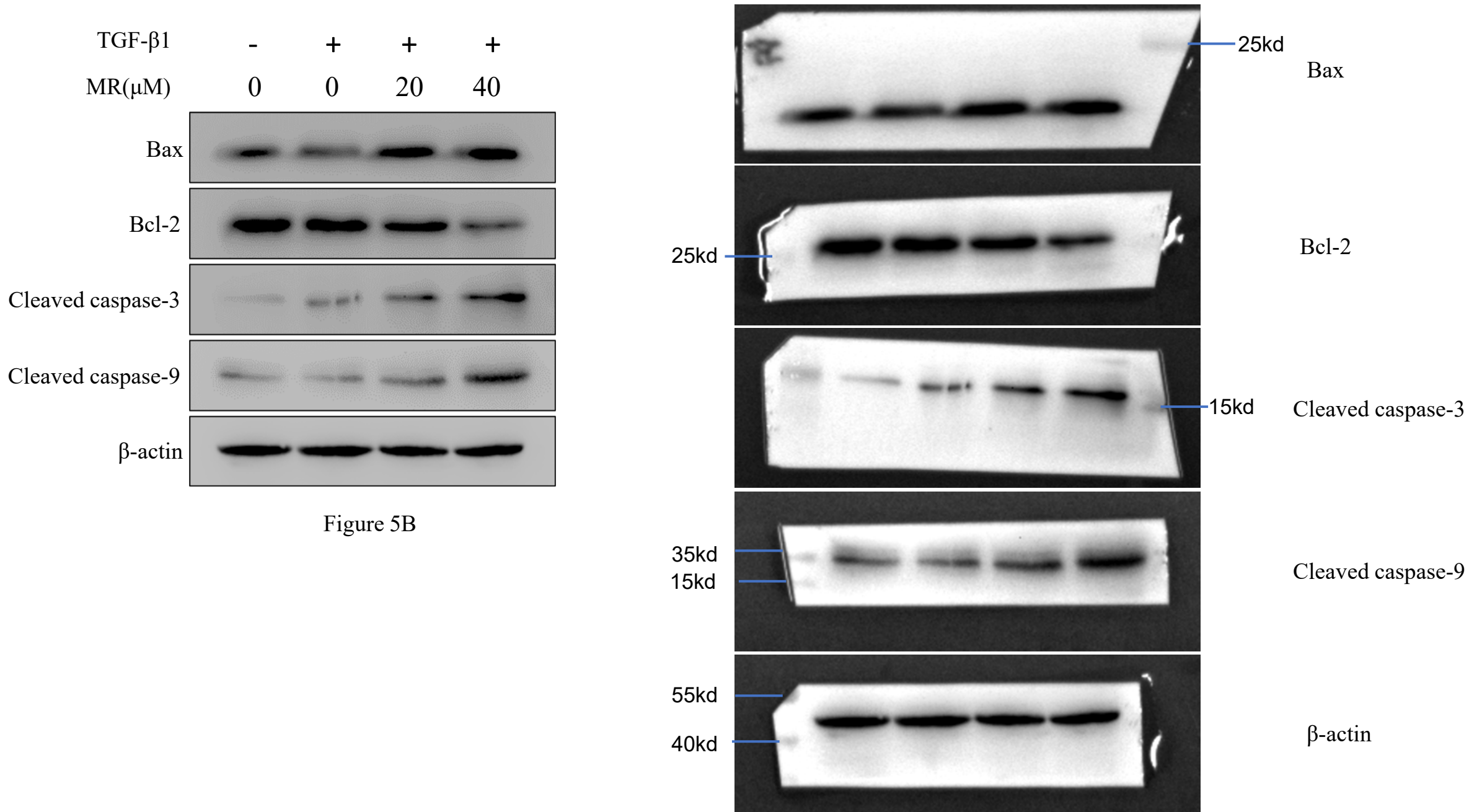

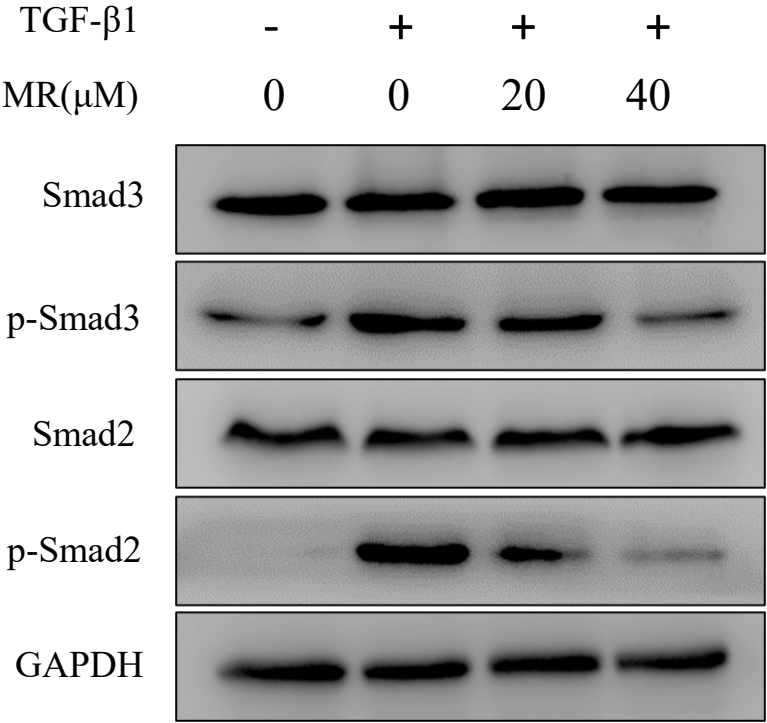

Figure 6A

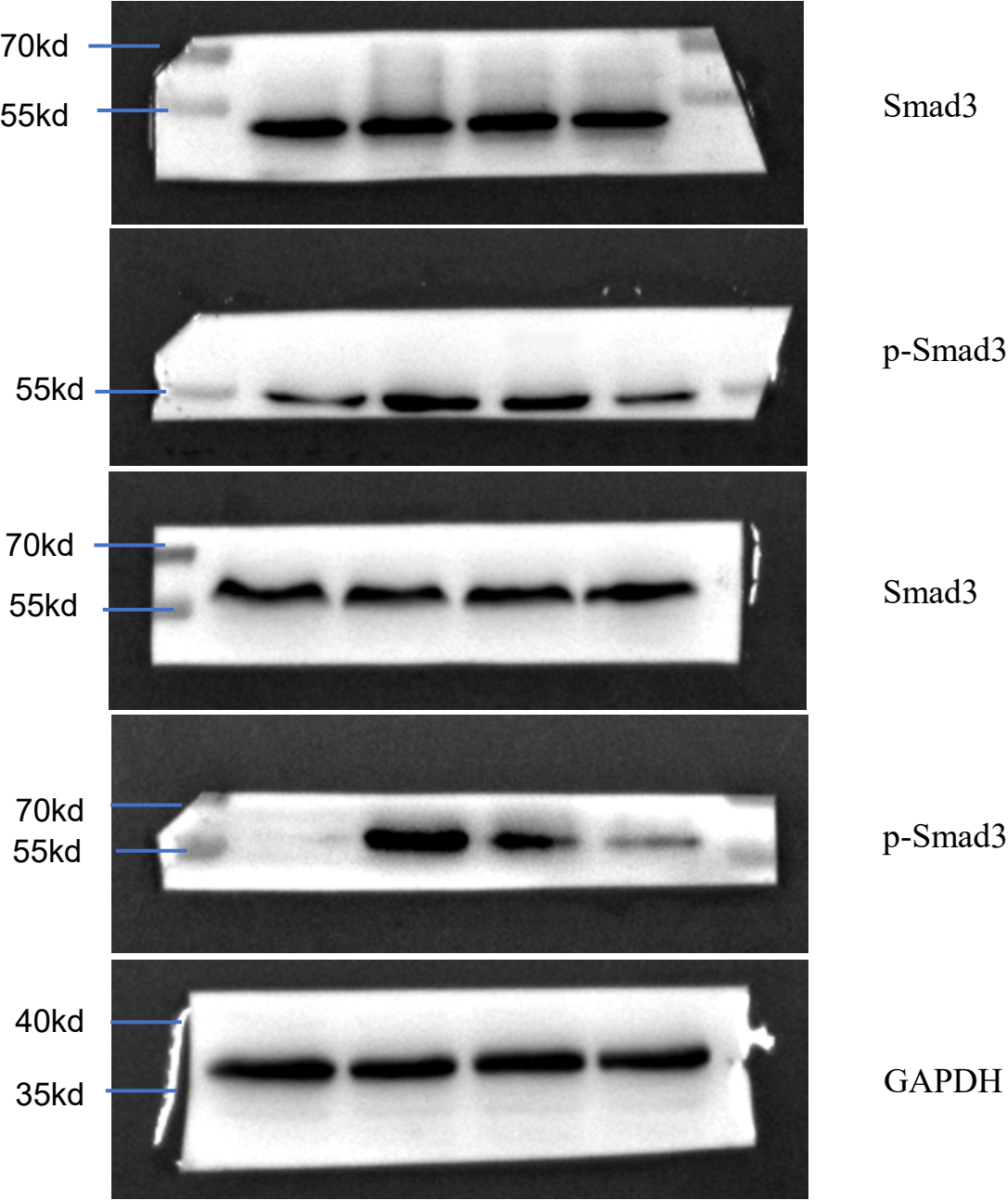

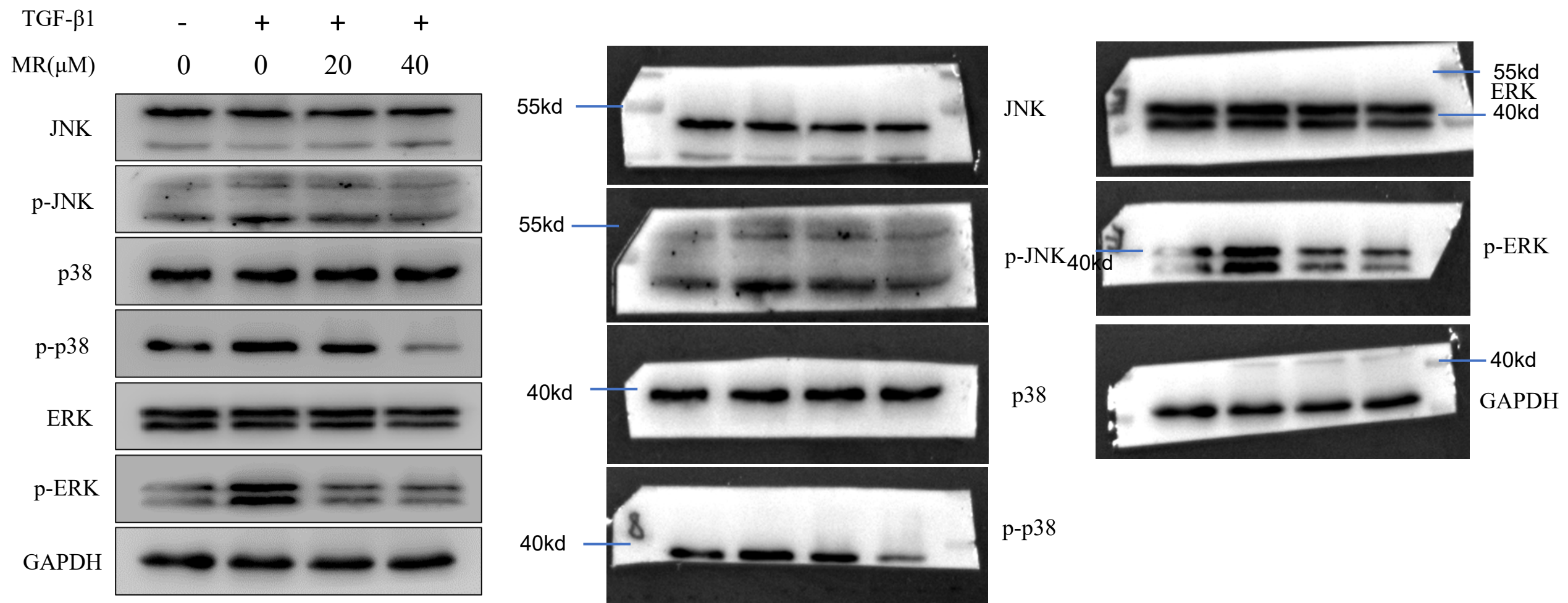

Figure 6C

Supplement: Supplementary file 1 [file DataSheet1.PDF]
